# EXPOSURE TO EXTREMELY LOW-FREQUENCY MAGNETIC FIELDS IN LOW- AND MIDDLE-INCOME COUNTRIES: AN OVERVIEW

**DOI:** 10.1093/rpd/ncaa172

**Published:** 2020-11-24

**Authors:** Dan Baaken, Daniel Wollschläger, Theodoros Samaras, Joachim Schüz, Isabelle Deltour

**Affiliations:** International Agency for Research on Cancer (IARC/WHO), Section of Environment and Radiation, 150 Cours Albert Thomas, 69372 Lyon CEDEX 08, France; Institute of Medical Biostatistics, Epidemiology and Informatics (IMBEI), University Medical Center Mainz, Langenbeckstraβe 1, 55131 Mainz, Germany; Institute of Medical Biostatistics, Epidemiology and Informatics (IMBEI), University Medical Center Mainz, Langenbeckstraβe 1, 55131 Mainz, Germany; School of Physics, Aristotle University of Thessaloniki, Thessaloniki 541 24, Greece; International Agency for Research on Cancer (IARC/WHO), Section of Environment and Radiation, 150 Cours Albert Thomas, 69372 Lyon CEDEX 08, France; International Agency for Research on Cancer (IARC/WHO), Section of Environment and Radiation, 150 Cours Albert Thomas, 69372 Lyon CEDEX 08, France

## Abstract

To compare extremely low-frequency magnetic field (ELF-MF) exposure in the general population in low- and middle-income countries (LMICs) with high-income countries (HIC), we carried out a systematic literature search resulting in 1483 potentially eligible articles; however, only 25 studies could be included in the qualitative synthesis. Studies showed large heterogeneity in design, exposure environment and exposure assessment. Exposure assessed by outdoor spot measurements ranged from 0.03 to 4μT. Average exposure by indoor spot measurements in homes ranged from 0.02 to 0.4μT. Proportions of homes exposed to a threshold of ≥0.3μT were many times higher in LMICs compared to HIC. Based on the limited data available, exposure to ELF-MF in LMICs appeared higher than in HIC, but a direct comparison is hampered by a lack of representative and systematic monitoring studies. Representative measurement studies on residential exposure to ELF-MF are needed in LMICs together with better standardisation in the reporting.

## INTRODUCTION

The International Agency for Research on Cancer (IARC) classified exposure to extremely low-frequency magnetic fields (ELF-MF) as possibly carcinogenic to humans (group 2B) in 2001^(1)^; this assessment has been more recently confirmed by the European Commission’s Scientific Committee on Emerging and Newly Identified Health Risks (SCENIHR)^(2)^. The IARC and SCENIHR classifications are mainly based on epidemiological studies of childhood leukemia. So far, experimental studies on animals failed to convincingly confirm an increased risk for leukemia^(3)^, and no plausible biophysical mechanisms have yet been identified^(4,5)^. A potential risk of ELF-MF for childhood leukemia has been investigated in high-income countries (HICs) in numerous studies in over 30 years of research^(6)^, two pooled analyses from 2000 showed significantly increased risks when children exposed to daily average ELF-MF of ≥0.4 μT^(7)^ and ≥0.3 μT^(8)^ were compared to children in the reference group with daily average exposure ≤0.1 μT. For children exposed to ≥0.4 μT, the relative risk was 2.0 (95% confidence interval (CI) 1.3–3.1)^(7)^ and for children exposed to ≥0.3 μT, the odds ratio (OR) was 1.7 (95% CI 1.2–2.3)^(8)^.

The most recent pooled analysis, which included studies published between 2000 and 2010 also suggested an increased risk for children exposed to daily average ELF-MF levels of ≥0.3 μT (OR 1.44, 95% CI 0.88–2.36), although not statistically significant^(9)^. Studies included in the mentioned pooled analyses were all from HICs like Germany^(10)^, UK^(11)^ or Sweden^(12)^, with the exception of data from one study from Brazil^(13)^. One percent or less of the children were categorised in the highest exposure group in the pooled analysis of Ahlbom and colleagues^(7)^. This shows that exposure to higher levels of ELF-MF is uncommon in HICs. Due to a different state of technical development, housing conditions and legal regulations, exposure levels in the general population and children in low- and middle-income countries (LMICs) could be different. In addition, LMICs typically have a higher proportion of children in the population. Taken together, more children in LMICs compared to HICs may be exposed to higher levels of ELF-MF as a potential risk factor to leukemia. Therefore, it is key to collect high-quality information on exposure to ELF-MF in these countries.

Although of the worldwide 1.958 billion children (age 0–14 years old), 1.758 billion live in LMICs^(14)^, we were not aware of any overview of exposure levels to ELF-MF in LMICs in the general population including children. For this reason, we compiled the first overview of studies from LMICs by applying techniques of a systematic review. In the following, we describe their main characteristics and results and discuss our findings in the context of results from measurement surveys conducted in HIC.

## MATERIAL AND METHODS

### Eligibility

All studies listed in PubMed and Web of Science (WoS) which included information on exposure to ELF-MF and reported on LMICs were eligible for inclusion. Specifically, we included studies reporting on exposure to ELF-MF with frequencies up to 300 Hz^(1)^, which reported measured or calculated magnetic fields for the general population, for children or for areas close to residential areas. Studies reporting on distance to power lines or substations as an exposure metric were also included. We excluded studies on occupational exposure or exposure from specific devices, cars or trains. We did not apply any restrictions in terms of health outcomes, study design, language, study size or time period. LMICs were defined in accordance with the definition of the World Bank (WB), based on the gross national income per capita in 2018. The LMICs were classified into three groups: low-income countries, lower middle income countries and upper middle income countries, as defined by the WB^(15)^.

### Search strategy

Electronic search was performed in PubMed and WoS. For the search in PubMed and WoS, we used a combination of keywords for ELF-MF and the name of each LMICs. A more detailed description of the search terms is included in ‘Supplementary A1’.

In addition to the systematic approach of the searches in PubMed and WoS, we used further extensive search techniques to identify all potentially relevant articles for this review: we conducted an informal survey among WHO-experts for electromagnetic fields (using a respective email distribution list) whom we asked for studies relevant to our research question; we included ‘snowballing’ methods, that is, screening the reference lists of the included studies for additional relevant articles, and; we systematically examined the issues of the last 2 years of the journal that published the majority of the included articles.

### Study selection and data extraction

After removing duplicates, all titles and abstracts identified by the search were screened for relevance. Full texts of potentially eligible articles were reviewed for inclusion. Studies fulfilling all eligibility criteria were included. Study selection was done by one reviewer (DB).

Relevant data were extracted by one reviewer (DB) using a predefined data extraction form, recording information on the author, year of publication, country, study type, information on measurement device and exposure assessment technique, and major results of the exposure assessment. When needed, ELF-MF units were converted from Gauss to Tesla, and if results were only provided graphically, values were abstracted from graphs, whenever possible^(16,17)^.

## RESULTS

### Study selection

**Figure 1:**
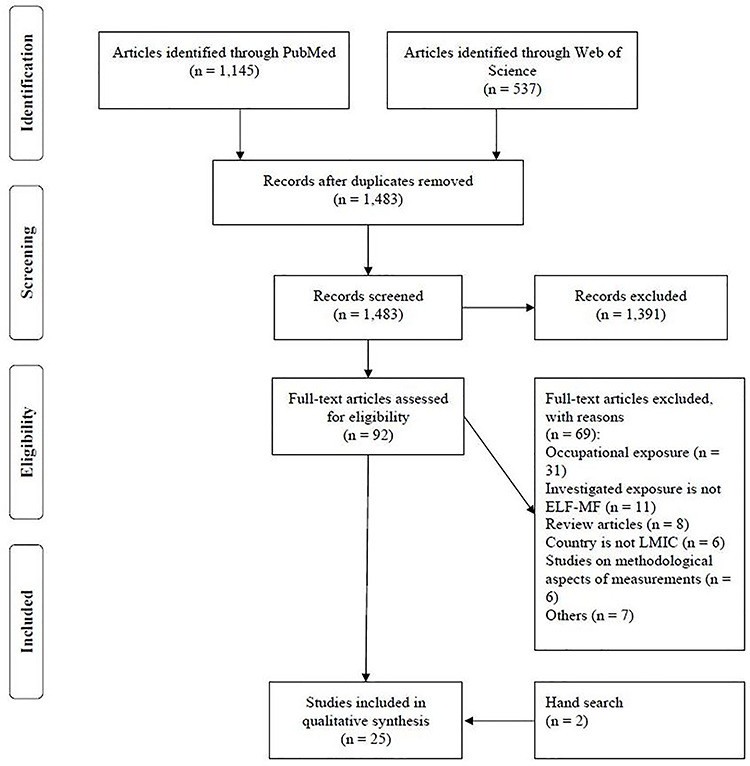
PRISMA flow diagram of study selection.

The systematic search in PubMed and WoS was done in October 2019. The flowchart for the process of study selection is presented in Figure 1. After removing duplicates from the initial search, 1483 articles were selected for the title and abstract screening, out of which 92 articles were retained for full-text screening. Evaluation of the 92 full texts left 23 original studies. Reasons for exclusion of full texts were: occupational exposure (*n* = 31), investigated exposure was not ELF-MF (*n* = 11), review articles not on exposure in LMICs (*n* = 8), the country the study was conducted in did not belong to LMICs (*n* = 6), studies on methodological aspects of measurements (*n* = 6) or other reasons (*n* = 7). Four of these latter seven articles reported on measurements in cars, trains or of specific devices, two on simulation studies and one on an in-vitro experimental study.

We added two articles that we identified through our additional extensive searches. One of these^(18)^ was identified by checking the issues of the last 2 years (November 2017–October 2019) of the journal *Radiation Protection Dosimetry*. The other article has been obtained through an informal survey among experts in the field^(19)^. Therefore, our overview included a total of 25 studies.

### Descriptive characteristics of the included studies

The 25 included articles were published between 1993 and 2019. The majority of the studies were published between 2015 and 2019 (*n* = 11)^(16,18–27)^ and 2010 and 2015 (*n* = 9)^(13,17,28–34)^. Three articles were published between 2005 and 2010^(35–37)^, one article was published in 1998^(38)^ and one in 1993^(39)^. The studies were conducted mainly in upper middle income countries (*n* = 22). From these 22 studies, the countries with the most contributions were China (*n* = 4)^(21,28,29,35)^ and Iran (*n* = 4)^(27,31,34,37)^. The origin of the other studies from upper middle income countries is displayed in Table 1. Four studies were conducted in lower middle income countries, specifically India^(30)^, West-Bank and Gaza^(18,19)^ and Tunisia^(16)^. We did not identify any study conducted in a low-income country (LIC). The identified studies are epidemiological studies (*n* = 12) and exposure studies (*n* = 14), which we defined as studies that reported measured or calculated ELF-MF exposure without assessing its potential impact on health outcomes. The epidemiological studies comprised three case studies^(27,35,38)^, two cross-sectional studies^(28,36)^, four case-control studies^(13,31,37,39)^ and three cohort studies^(21,29,34)^. Various health outcomes were considered in the epidemiological studies. The most frequent was cancer (*n* = 5) with four studies on childhood leukemia^(13,35,37,39)^ and one study on cancer in young adults^(38)^, other studies analyzed fetal^(21)^ or child growth^(34,36)^. neurobehavioural function^(28)^, memory loss in children^(27)^ and risk of miscarriage^(29,31)^. The exposure studies consisted of 13 studies with indoor or outdoor measurements of exposure of ELF-MF and one study with calculated exposure based on data of power lines^(22)^ (Table 1). The voltage levels of the power lines differed between the included studies, namely 66 kV^(19)^, 90 kV^(16)^, 150 kV^(16)^, 154 kV^(26)^, 220 kV^(20)^, 225 kV^(16)^ and 400 kV power lines^(22,30)^. One exposure study conducted in India^(30)^ reported results of measurements inside a substation, in addition to measurements of 400 kV transmission lines. Since a substation should only be accessible to persons working there and not the general population, we did not include the results of these specific measurements in our overview.

**Table 1:** Overview of studies conducted in low- and middle-income countries, reporting on exposure to ELF-MF, sorted by country.

Authors (Year)	Country	Study type	Exposure assessment method	Reporting of levels of magnetic flux density
Tourab & Babouri (2016)^(20)^	Algeria	Exposure study	Outdoor spot measurements	Yes
Wunsch-Filho *et al*. (2011)^(13)^	Brazil	Case-control study	Outdoor & indoor spot measurements	Yes
Koifman *et al*. (1998)^(38)^	Brazil	Case study	Outdoor & indoor spot measurements	Yes^a^
Zaryabova *et al*. (2013)^(17)^	Bulgaria	Exposure study	Indoor spot measurements	Yes^b^
Ren *et al*. (2019)^(21)^	China	Cohort study	24-h personal measurements	Yes
Huang *et al*. (2013)^(28)^	China	Cross-sectional study	Outdoor and indoor spot measurements not reported separately	Yes
Wang *et al*. (2013)^(29)^	China	Cohort study	Outdoor spot measurements	Yes
Yang *et al*. (2008)^(35)^	China	Case study	Outdoor spot measurements	Yes
Fadel *et al*. (2006)^(36)^	Egypt	Cross-sectional study	Calculated exposure	No
Aravind *et al*. (2014)^(30)^	India	Exposure study	Outdoor spot measurements	Yes^b^
Ghadamgahi *et al*. (2016)^(27)^	Iran	Case study	Indoor spot measurements	Yes
Mahmoudabadi *et al*. (2013)^(31)^	Iran	Case-control study	Indoor spot measurements	Yes^a^
Mahram & Ghazavi (2013)^(34)^	Iran	Cohort study	Outdoor spot measurements	Yes^a^
Feizi & Arabi (2007)^(37)^	Iran	Case-control study	Calculated exposure	Yes
Fajardo-Gutierrez *et al*. (1993)^(39)^	Mexico	Case–control study	Distance to source	No
Lunca *et al*. (2018)^(22)^	Romania	Exposure study	Calculated exposure	Yes
Ursache *et al*. (2016)^(23)^	Romania	Exposure study	Outdoor & indoor spot measurements	Yes
David & Nica (2012)^(32)^	Romania	Exposure study	Indoor spot measurements	Yes
Vulevic & Osmokrovic (2011)^(33)^	Serbia	Exposure study	Outdoor & indoor spot measurements	Yes
Rathebe *et al*. (2018)^(24)^	South Africa	Exposure study	Outdoor spot measurements	Yes
Silangam *et al*. (2017)^(25)^	Thailand	Exposure study	Indoor spot measurements	Yes
Ghnimi *et al*. (2018)^(16)^	Tunisia	Exposure study	Outdoor spot measurements	Yes^b^
Carlak *et al*. (2017)^(26)^	Turkey	Exposure study	Outdoor spot measurements	Yes
Abuasbi *et al*. (2018)^(18)^	WBG	Exposure study	Indoor spot measurements	Yes
Abuasbi *et al*. (2018)^(19)^	WBG	Exposure study	Outdoor spot measurements	Yes

*Note:* WBG = West Bank and Gaza.

^a^Authors reported exposure in Gauss.

^b^Relevant measurements for magnetic flux density only reported in figures.

For the remainder of our overview, we considered separately studies with personal measurements (*n* = 1), with spot measurements (outdoor spot measurements (*n* = 8), indoor spot measurements (*n* = 6) and studies that reported separately on both (*n* = 4)) and studies with other exposure assessment methods (*n* = 4) (Table 1). One study^(28)^ with indoor and outdoor measurements was excluded from assignment to these categories as the authors did not report separate results from the measurement of indoor and outdoor exposure, and another study was excluded because we could not extract the exposure data from the figures with the required accuracy^(30)^.

### Personal measurements

One study reported 24-h personal measurements for 128 pregnant women in China^(21)^. Measurements were carried out using the EMDEX Lite meter as a measurement device, located at the waist in the daytime and next to the beds while sleeping. Measurements were taken every 4 s. A median of the time-weighted average of 0.06 μT was observed in these women.

### Outdoor spot measurements

Characteristics and major findings of all 12 studies with reported measurements of outdoor exposure to ELF-MF are displayed in Table 2. Four studies^(16,19,20,26)^ reported measured values in the vicinity of power lines of different voltages ranging from 66 to 400 kV measured at 1 m above the ground. The results of the measurements ranged from 0.03 μT for a measurement of a 90 kV double-circuit power line^(16)^ to 3.5 μT observed in the vicinity of a 66 kV power line^(19)^. Four studies^(13,28,33,35)^ reported exposure measurements outside the homes of participants. In the study of Wunsch-Filho *et al*.^(13)^, 14.8% of the measurements at the front door were above 0.3 μT, while 13.3% of the measurements from Wang *et al*.^(29)^ were above 0.4 μT at the front door. The other two studies^(33,35)^ reported measured values outside the house depending on the distance to power lines. A house located 24 m away from a 400 kV power line yielded the highest measured value of 4 μT^(33)^. In studies on exposure of the general public in residential areas^(23,24,34,38)^, the mean exposure ranged from 0.15 μT obtained in a series of 74 outdoor measurements in an urban area in Romania^(23)^ to 2.18 μT observed below a 500 kV power line in a village of a rural area in Brazil^(38)^. Three studies used the same measurement device^(16,33,34)^, while the rest of the studies used different devices (Table 2).

**Table 2:** Studies from LMICs with outdoor spot measurements of ELF-MF close to power lines, houses and residential areas: exposure assessment and major findings.

Authors	Exposure environment and measurement	Measurement device	Major findings
Power lines			
Tourab & Babouri^(20)^	Exposure environment: in the vicinity of 220 kV power lines (*n* = 2) in a city. Measurements taken at 0, 1, 1.5 and 1.8 m above ground level at different distances of the lines.	Electromagnetic field meter PM8053B	Measurements 1 m above ground under 2 power lines at different positions: 2.58–2.74 μT measurements 1 m above ground for single power line at different positions: 2.20–2.53 μT
Ghnimi *et al*.^(16)^	Exposure environment: under 90, 150 and 225 kV single-circuit and double-circuit power lines in the vicinity of a transformer station. Measurements made at 1 m above ground at various times of the day and distances with a max. distance of 50 m.	HI-3604 ELF survey meter	Measurements^a^ of magnetic field at various distances from the lines. For a single-circuit power line near a transformer station: 90 kV: 0 m: 0.4 μT; 25 m: 0.25 μT; 50 m: 0.1 μT 150 kV: 0 m: 0.1 μT; 25 m: 0.95 μT; 50 m: 0.1 μT 225 kV: 0 m: 0.05 μT; 25 m: 0.35 μT; 50 m: 0.07 μT For a single-circuit power line far away from a transformer station: 90 kV: 0 m: 0.05 μT; 25 m: 0.15 μT; 50 m: 0.07 μT 150 kV: 0 m: 0.05 μT; 25 m: 0.28 μT; 50 m: 0.1 μT 225 kV: 0 m: 0.05 μT; 25 m: 0.15 μT; 50 m: 0.05 μT For a double-circuit power line near a transformer station: 90 kV: 0 m: 0.03; 25 m: 0.18; 50 m: 0.05 µT 150 kV: 0 m: 0.38; 25 m: 0.1; 50 m: 0.1 µT 225 kV: 0 m: 0.58; 25 m: 0.65; 50 m: 0.45 µT For a double circuit power line far away from a transformer station: 90 kV: 0 m: 0.1; 25 m: 0.15; 50 m: 0.1 µT 150 kV: 0 m: 0.1; 25 m: 0.1; 50 m: 0.1 µT 225 kV: 0 m: 2.70; 25 m: 0.45; 50 m: 0.43 µT
Carlak *et al*.^(26)^	Exposure environment: in the vicinity of 154 kV power lines in an urban area. Measurements taken at 0.2, 0.5, 1, 1.5 and 2 m above ground level.	Hitester 3470	Measurements 1 m above ground and various distances to power lines: 0 m: 1.65 μT, 10 m: 1.32 μT, 20 m: 0.75 μT, 30 m: 0.41 μT, 40 m: 0.25 μT
Abuasbi *et al*.^(19)^	Exposure environment: in the vicinity of 66 kV power lines (n = 40 lines). Measurements taken at 1 m above the ground over a 6 min period.	Spectrum Analyzer NF-5035	Max. and min. mean values: 3.5 μT, 0.89 μT
Outsides houses			
Wunsch-Filho *et al*.^(13)^	Exposure environment: outside the front door of homes with children. Measurements of 3 min duration.	EMDEX-II dosemeter	Distribution of exposure levels of all measurements: <0.1 μT: 50.4% 0.1–0.3 μT: 34.8% ≥0.3 μT: 14.8%
Wang *et al*.^(29)^	Exposure environment: outside the front doors and in the alleys of 552 homes of pregnant women on days with relatively high power supply loads. Measurements (*n* = 5) of 16 s min. duration.	EFA-300 electric and magnetic field analyzers	Median (min.– max.) exposure at different locations: front door of residence: 0.098 μT (0.012 μT–2.04 μT) alley of the residence: 0.099 μT (0.012 μT–4.26 μT) distribution of exposure levels of all measurements: ≤0.05 μT: 33.6% >0.4 μT: 13.3% >1.0 μT: 4.6%
Yang *et al*.^(35)^	Exposure environment: residential areas of 66 cases of childhood leukemia with information on distance to electric transformers and power lines.	EMF detector TriField meter	Mean peak values at various distances to electric transformers and power lines: 50 m: 0.18 μT (*n* = 9), 100 m: 0.14 μT (*n* = 13), 500 m: 0.13 μT (*n* = 19)
Vulevic & Osmokrovic^(33)^	Exposure environment: under or between 110, 220 and 400 kV power lines in yards of 35 municipalities. Measurement were performed under or between the power lines at height of 1.8 m over ground.	HI-3604 ELF survey meter	Maximum values: at 12.6 m distance from 110 kV line: 2 μT at 8.3 m distance from 220 kV line: 2.5 μT, at 24 m distance from 400 kV line: 4 μT
Residential areas			
Rathebe *et al*.^(24)^	Exposure environment: residential areas near substations. Measurements at 1 m above the ground for 0, 3, 6 and 9 m distances to substations.	TriField meter model XE 100	Mean, range, and SD at various distances between substation and residential area: 0 m: mean 0.62 μT, range 0–1.70 μT, SD 0.28 μT 3 m: mean 0.30 μT, range 0–1.20 μT, SD 0.16 μT 6 m: mean 0.22 μT, range 0–0.84 μT, SD 0.15 μT 9 m: mean 0.16 μT, range 0–1.80 μT, SD 0.24 μT
Koifman *et al*.^(38)^	Exposure environment: Amazon village near 500 kV power lines. Measurements at 1 m above the ground taken during community activities near power lines: ritual races, cattle herding, activities below transmission lines.	AMEX meter	Cumulative and mean exposure during different activities: ritual race 0.2 μT/h; 0.35 μT cattle herding 2.43 μT/h; 0.68 μT below transmission line at day I: 1.34 μT/h; 1.99 μT, at day II: 18.1 μT/h; 2.18 μT
Mahram & Ghazavi^(34)^	Exposure environment: a city northwest of Tehran. Measurements taken around high-voltage lines and for ‘control’ areas that were 2–3 streets away.	HI-3604 ELF survey meter	Measurements under lines or up to 25 m distance: mean: 0.31 μT ± 0.18 μT, max.: 0.50 μT ‘control’ areas: mean: 0.04 μT ± 0.004 μT, max.: 0.10 μT
Ursache *et al*.^(23)^	Exposure environment: along a street close to apartment buildings in locations in the vicinity of a substation and below a high-voltage power line. Measurements at height of 1 m above the ground and at ≥1 m away from any source (*n* = 74).	Hand-held, triple axis 480826 EMF tester	Measurements around the substation: min.: 0.09 μT, max.: 0.30 μT, mean: 0.16 μT mean of all 74 outdoor measurements: 0.15 μT

*Notes*: max. = maximum; min. = minimum.

^a^Results presented on the original report only graphically, numbers reported here were extracted from the figures by DB and ID.

### Indoor spot measurements

We described the characteristics and major findings of the studies with indoor spot measurements (*n* = 10) in Table 3. In most of the studies, measurements were performed inside homes (*n* = 7). Two studies reported on measurements inside schools^(25,27)^ and one study on exposure during housekeeping, most likely an indoor activity^(38)^. Two studies^(13,18)^ with measurements in homes provided information on the distribution of the measured ELF-MF exposure values: 24-h measurements under children’s beds showed that 6.19% of 727 measurements were ≥0.3 μT^(13)^. In a study with measurements in apartments in buildings under normal power use, 19% were >0.1 μT and 13% between 0.3–0.4 μT^(18)^. Average values of all studies with indoor measurements in homes ranged from 0.02^(23,32)^ to 0.4 μT, which was observed in houses of women with unexplained spontaneous abortions in Iran^(31)^. There were different settings for the exposure assessment between the studies with measurements in homes including measurements in homes near substations^(23)^, homes under 110 kV, 220 kV and 400 kV power lines^(33)^ or measurements in apartment buildings with built-in transformer stations or indoor power substations^(17,18)^. The study on apartment buildings with built-in transformer station measured an average exposure of ~0.28 μT at 1 m above the floor in apartments directly above or next to the transformer with maximum values of 0.65 μT at 0.5 m height^(17)^. Other studies with maximum exposure measurements exceeding 0.4 μT reported maximum values of 0.45 μT in a residence with an indoor power substation^(18)^ and 3.2 μT for a house under a 400 kV power line^(33)^. One of the two studies on measurements in schools reported on ELF-MF depending on the distance to substations^(27)^. Schools close to substations (30–50 m) showed higher average exposure levels compared to schools far away from substations (610–1390 m) (Table 3). Mean exposure of measurements in 60 classrooms in Bangkok^(25)^ was 0.11 μT, with 21.67% of the classrooms having an exposure level above 0.2 μT. Only two studies used the same measurement device, the EMDEX-II dosemeter^(13,17)^.

**Table 3:** Description of exposure assessment and results of studies from LMICs with indoor spot measurements on ELF-MF in homes, in schools and in residential areas.

Authors	Exposure environment and measurement	Measurement device	Major findings
Homes			
Zaryabova *et al*.^(17)^	Exposure environment: randomly selected buildings with built-in transformer stations in regions of Sofia. Measurements done in 65 apartments of 21 buildings with apartments directly above and next to transformer (type A), in the same building randomly selected apartments in over floors (type B) and apartments on the same floor as type A (type C). Measurements performed at centre of each room and 1.4 away from corners of the room in 0.5 and 1 m height. Additional measurements were performed.	Not reported	Averages^a^ for type A apartments: ~0.35 (approx. min. 0.1–max. 0.65) at 0.5 m height ~0.28 (approx. min. 0.13–max. 0.57) at 1 m height averages^a^ type B apartments: ~0.25 (approx. min. 0.09–max. 0.5) at 0.5 m height ~0.21(approx. min 0.09–max. 0.48) at 1 m height averages^a^ for type C apartments: ~0.1 (approx. min 0.05–max. 0.25) at 0.5 m height ~0.06 (approx. min 0.03–max. 0.28) at 1 m height
Wunsch-Filho *et al*.^(13)^	Exposure environment: each room of a house with children living in it (*n* = 727). Measurements for 24 h under the child’s bed.	EMDEX-II dosemeter	Distribution of exposure levels for 24-h measurements: <0.1 μT: 69.74% 0.1 μT–<0.3 μT: 24.07% ≥0.3 μT: 6.19%
Mahmoudabadi *et al*.^(31)^	Exposure environment: homes. Measurements in homes of 58 women with spontaneous abortion and 58 pregnant women.	3D EMF tester Model ELF-828	Mean value in houses of women with unexplained spontaneous abortion: 0.4 μT ± 0.31 μT mean value in houses of pregnant women: 0.14 μT ± 0.15 μT
Ursache *et al*.^(23)^	Exposure environment: three apartments near substations. Measurements at height of 1 m above the ground and at ≥1 m away from any source in normal operating conditions of existing appliances over 24 h with time intervals of 1 h.	Hand-held, triple axis 480826 EMF tester	Mean value in apartment 1 (in vicinity of a power substation): 0.08 μT mean value in apartment 2 0.02 μT mean value in apartment 3 0.02 μT
David & Nica^(32)^	Exposure environment: One apartment in a residential area. Measurements taken for 1 h in one room every 10 s.	Measurement device developed by the authors	Root mean square values: min.:0.01 μT max.: 0.04 μT average: 0.02 μT (SD 0.01 μT)
Vulevic & Osmokrovic^(33)^	Exposure environment: homes in 35 municipalities under 220 and 400 kV power lines. Measurements in the middle of the living and sleeping rooms at 1.5–1.8 m above the floor.	HI-3604 ELF survey meter	Max. values in houses: under 400 kV power line (height 15 m over ground) in the living room 3.2 μT under 220 kV power line (height 20 m over ground) in the sleeping room 3 μT
Abuasbi *et al*.^(18)^	Exposure environment: 32 residences distributed randomly over the city of Ramallah, including apartments within buildings, detached houses and few buildings with indoor power substations. Measurements conducted at zero-power use and normal power use at 1 m above the floor for 6 min in living rooms or bedrooms.	Spectrum Analyzer NF-5035	Under normal power use: max.: 0.45 μT distribution of values: <0.1 μT: 81% >0.1 μT: 19% 0.3 μT–0.4 μT: 13%
Schools			
Ghadamgahi *et al*.^(27)^	Exposure environment: 2 schools close to substations (school A: 30 m, school B: 50 m) and 2 schools far away from substations (school C: 610 m, school D 1390 m). Measurements (*n* = 200) were done 1 m above ground in accordance to the IEEE standard procedures.	3-axis Gauss meter model TES-1394	School A: classroom 0.25 μT, corridor: 0.27 μT, courtyard: 0.34 μT μT, average: 0.28 μT School B: classroom 0.19 μT, corridor: 0.20 μT, courtyard: 0.23 μT, average: 0.21 μT School C: classroom 0.16 μT, corridor: 0.16 μT, courtyard: 0.18 μT, average: 0.17 μT School D: classroom 0.16 μT, corridor: 0.16 μT, courtyard: 0.17 μT, average: 0.16 μT
Silangam *et al*.^(25)^	Exposure environment: 60 classrooms of three secondary schools in Bangkok. Measurement during class hours (Monday–Friday between 8:30–16:30) done at five points in the classroom (centre of the room and at the four corners) at 1 m above the floor. Average measurement time was 6 min.	EFA-300 Field Analyzer	Median (min.–max.), mean (SD) value for all schools median 0.09 μT (min. 0.001 μT −0.42 μT) mean 0.11 μT (SD 0.10 μT) 21.67% of classrooms >0.2 μT
Residential areas			
Koifman *et al*.^(38)^	Exposure environment: Amazon village near 500 kV power lines. Measurements taken during housekeeping at 1 metre above the ground.	AMEX meter	Cumulative and mean exposure during housekeeping: 0.23 μT/h; 0.07 μT

^a^Results presented on the original report only graphically, numbers reported here were extracted from the figures by DB and ID.

### Other types of exposure assessment

Three studies calculated exposure estimates^(22,36,37)^. Feizi & Arabi^(37)^ investigated the potential risk of 123, 230 and 400 kV power lines on acute childhood leukemia in Iran. They calculated the exposure based on the mean intensity of the electrical current and additional line characteristics for 119 children of whom 16 lived in a distance of ≤500 m to a power line. In total, 16.8% of all children were exposed ≥0.45 μT and 83.2% were exposed to <0.45 μT. Lunca *et al*.^(22)^ calculated the ELF-MF from 400 kV overhead power lines with the software tools PowerMag and PowerELT. They report various results of calculated magnetic flux density stratified for single-circuit lines and double-circuit lines. They conclude that the typical levels under 400 kV single-circuit power lines at 1 m above the ground level are 5 μT and under double-circuit power lines, 4.5 μT. Fadel *et al*.^(36)^ compared 390 children living <50 m close to a power line with a control group of 390 children from another area in Egypt. Although the authors stated that they calculated the exposure, they did not report results for the calculations. One study in Mexico assessed the distance to transformers, high-voltage power lines and electric substations as an approximation for the exposure to ELF-MF to investigate the risk of childhood leukemia^(39)^.

## DISCUSSION

### Brief summary

The goal of our review was to give the first comprehensive literature overview on exposure to ELF-MF in the general population including children of LMICs by applying methods of a systematic review. We identified 25 studies published between 1993 and 2019 in total, consisting of 21 studies from upper middle income countries, four studies from lower middle income countries and not a single study from LIC. Eighteen studies reported extractable results of spot measurements to estimate the exposure to ELF-MF using outdoor spot measurements (*n* = 8), indoor spot measurements (*n* = 6) or both (*n* = 4). The included studies showed a large heterogeneity in their design, exposure environment, exposure assessment and reported summary statistics, which severely hampered their comparability. Single outdoor spot measurements for ELF-MF ranged from 0.03 μT to a maximum of 4 μT. Average exposure from indoor spot measurements in homes ranged from 0.02 to 0.4 μT.

### Comparison with results from HICs

For our comparison of studies from LMICs and HIC, we did not take into account studies that limited their exposure measurements to specific power lines with no reference to proximity to residential areas. Also, we described these studies earlier because the values reported in those studies and measured under power lines could give an idea of the potential exposure in the respective countries, when close to residential areas.

In a review, the WHO estimated the exposure to residential ELF-MF in the USA and Europe^(40)^. In the USA, the geometric mean of the magnetic fields over one day ranged between 0.06 and 0.11 μT in homes. For Europe, consisting of HIC, the geometric mean of the magnetic fields was lower, estimated to be in the range of 0.03 to 0.07 μT. The WHO also reported the proportion of children being exposed above the thresholds of 0.3–0.4 μT, levels which are possibly associated with an increased leukemia risk in epidemiological studies:^(7,8)^ between 1 and 4% of children were estimated to have exposures ≥0.3 μT and 1–2% of children exposures above 0.4 μT in HIC. Another study assessing the potential health impacts of residential exposure to ELF-MF in Europe^(41)^ estimated the distribution of exposure to residential ELF-MF based on a literature overview. They reported that 0.54% of the general population was exposed to a geometric mean exposure of above 0.3 μT. A measurement survey on residential exposure to ELF-MF in Australia^(42)^ consisting of spot measurements in 296 randomly selected homes in Melbourne showed consistency with the estimates for the USA and Europe reported by the WHO. The average fields were 0.05–0.06 μT and exposure in 2% of the homes was above 0.4 μT. A survey in Taiwan^(43)^ in homes occupied by children under 7 years of age showed higher exposures compared to the USA, Europe and Australia. Spot measurements had been performed in 2214 randomly selected households. Mean magnetic fields were 0.121 μT and 7.3% of the included households were exposed to levels above 0.3 μT and 5.4% above 0.4 μT. However, the study from Taiwan reported results of single spot measurements that lasts about 30–40 s, while the studies from the USA, Europe and Australia reported results of 24-h measurements.

The WHO concluded that residential exposure to ELF-MF did not vary dramatically across the world^(40)^ in 2007. Our overview suggests that this is not necessarily generalizable to all places around the world, as it revealed exposure levels in LMICs exceeding the levels reported for the USA, Europe, Australia and Taiwan. In our systematic search, we identified studies from Brazil^(13)^, China^(29)^, Iran^(37)^ and the West Bank and Gaza^(18)^ in which larger proportions of persons had been exposed to residential ELF-MF above 0.3 μT or 0.4 μT. In the Brazilian study^(13)^, 14.8% of the spot measurements performed outside the front door resulted in ≥0.3 μT, and 6.19% was ≥ 0.3 μT for 24-h measurements inside the houses. In a Chinese study^(29)^, 13.3% of the measurements at the front door of homes of pregnant women were above 0.4 μT, with 4.6% exceeding 1 μT. The Iranian study^(37)^ reported that the calculated exposure to ELF-MF in 16.8% of 119 homes with children was above 0.45 μT. In a study of the West-Bank and Gaza^(18)^, a comparable high proportion of this exposure level was identified by indoor spot measurements (13% exposed to 0.3–0.4 μT) under normal power use.

The comparison of the results from LMICs with studies from HIC is only possible to a limited extent, since only the Brazilian study^(13)^ performed 24-h indoor spot measurements comparable to those in the USA, Europe and Australia. Single spot measurements as performed in the studies of West-Bank and Gaza^(18)^ and China^(29)^, therefore, can only be compared to the results of the study from Taiwan. Also, the Chinese study reported on outdoor spot measurements. Spot measurements are considered the simplest form of measurements as they are not able to capture the temporal variability during the day as well as between-day variability during e.g. a week or seasonal differences^(40)^. Twenty-four-hour measurements improve the assessment of temporal variability because short-term increases of magnetic fields by devices or wiring do not influence the average field^(41)^.

### Considerations for studies on ELF-MF in LMICs

We identified several methodological limitations in studies carried out in LMICs with respect to the conduct and the reporting of the studies. Applying standardised measurement routines and strengthening standardisation in reporting would be beneficial for future studies in LMICs and would allow for a better comparability with studies from HIC.

#### Conduct

Studies in LMICs could have been prompted by high exposure situations, providing a biased view of exposure levels. Nevertheless, some studies showed in fact that high exposure situations exist, but they have to be set in context, e.g. by showing how frequently these situations occur. Results should therefore be interpreted with caution and they cannot be generaliesd. In LMICs, permanent and reliable access to electricity is not always available for the whole population. In parallel to improvements of the electricity distribution networks, conducting measurement surveys drawn from a random sample must be a future goal. Therefore, studies should not solely include homes near substations, power lines and apartment buildings with transformer stations, but include randomly selected homes to obtain population-representative exposure. By conducting a pilot study, first, the feasibility of such an approach needs to be assessed in the local context. Results of the pilot study should also allow to perform power calculations^(42)^. Predefined measurement protocols for at least 24-h measurements should be developed and used. These protocols should include information about the measurement device and the calibrations for this device. The protocol should also give clear instructions on how to perform the measurement on-site (e.g. in the apartment). Besides measurements, authors should try to collect additional information that could have impacted the exposure, e.g. type of household, status of electric devices (on or off during measurements), temperature, measured distance to and voltage of power lines, presence and type of substations.

#### Reporting

A strengthening of standardisation of reporting could lead to a better comparison of the studies. Especially reporting on key elements of the exposure assessment is important, including but not limited to the measurement device, time and season of measurement and measurement techniques. The authors should, therefore, state if they used uniaxial or triaxial measuring probes, as well as which field values they report (total field, maximum along a single axis). They should also indicate whether the measurement device was calibrated according to manufacturer’s specifications. It is also important that results are reported in a comparable way, in tables. This should include reporting on average measured exposure of magnetic fields (at least arithmetic and geometric mean, standard deviation, and median and other percentiles) and distribution of exposure levels, with emphasis on the exposure levels higher than 0.3 μT and 0.4 μT.

### Strengths and limitations of the study

One strength of our study is that the process of literature search, screening and study selection was done systematically and in accordance with the Preferred Reporting Items for Systematic Reviews and Meta-Analyses (PRIMSA) workflow^(44)^. We included two different databases, performed the study selection by applying *a priori* defined eligibility criteria and extracted data in a predefined database.

Additional strengths of our overview are that we made extensive efforts to identify relevant publications on this topic by checking two electronic databases (PubMed and WoS) and complementing this by additional efforts, including the examination of the issues of the last 2 years of the journal that published the largest number of included studies, checking the reference lists of included articles for other relevant articles and conducting an informal survey among expert in the field of electromagnetic fields via an existing email list including experts who are collaborators of the WHO. These efforts confirmed that our search had been exhaustive, since we only identified two additional articles: both articles had been missed because of the rare situation that the official country name ‘West Bank and Gaza’ had not been used in the article which referred to the study location as ‘Palestine’.

Despite the strength, our study has some limitations. The objectivity in terms of selection and data extraction process could have been further improved by adding a second independent reviewer. Furthermore, we cannot rule out the possibility that we miss potential relevant articles by using search terms only in the English language.

## CONCLUSION

Data on exposure to ELF-MF in the general population of LMICs were sparse and even non-existent for LIC. Studies were heterogeneous and quality of reporting was limited. Direct comparison with systematic monitoring surveys from HIC is very limited with the data available. Some studies showed measured ELF-MF levels higher compared to studies from HIC, indicating a need for further analysis. However, the generalizability of the currently available evidence is unknown. There is an urgent need for future systematic studies with randomly drawn samples, sound measurement methods based on predefined protocols and standardised reporting, before any firm conclusions on ELF-MF exposure in the general population in LMICs can be drawn.

## Supplementary Material

Baaken_Appendix_RPD-20-0342_R1_ncaa172
