# Supplementary material for: EXPOSURE TO EXTREMELY LOW-FREQUENCY MAGNETIC FIELDS IN LOW- AND MIDDLE-INCOME COUNTRIES: AN OVERVIEW
Source: Radiat Prot Dosimetry. 2020 Nov 24;191(4):487–500. doi: 10.1093/rpd/ncaa172 (PMC7745074; doi:10.1093/rpd/ncaa172)
Supplement: Baaken_Appendix_RPD-20-0342_R1_ncaa172 [file baaken_appendix_rpd-20-0342_r1_ncaa172.docx]

**APPENDIX**

**Supplementary File 1: Search terms used in PubMed to identify studies on exposure to extremely low-frequency magnetic fields in LMIC**

| **PubMed** | |
| --- | --- |
|  | **Exposure** |
| **#1** | low frequency magnetic field OR ELF-MF OR ELF magnetic field |
|  | **Low income countries** |
| **#2** | Afghanistan OR Benin OR Burkina Faso OR Burundi OR Central African Republic OR Chad OR Congo, Dem. Rep OR Congo OR Eritrea OR Ethiopia OR Gambia, The OR Gambia OR Guinea OR Guinea-Bissau OR Haiti OR Korea, Dem. People's Rep. OR North Korea OR Liberia OR Madagascar OR Malawi OR Mozambique OR Nepal OR Niger OR Rwanda OR Sierra Leone OR Somalia OR South Sudan OR Syrian Arab Republic OR Syria Tajikistan OR Tanzania Togo OR Uganda OR Yemen, Rep. OR Yemen |
|  | **Lower-middle income countries** |
| **#3** | Angola OR Bangladesh OR Bhutan OR Bolivia Cabo Verde OR Cambodia OR Cameroon OR Comoros OR Congo, Rep. OR Congo OR Côte d'Ivoire OR Djibouti OR Egypt, Arab Rep. OR Egypt OR El Salvador OR Ghana OR Honduras OR Indonesia OR Kenya OR Kiribati OR Kyrgyz Republic OR Kyrgyzstan OR Lao PDR OR Laos OR Lesotho OR Mauritania OR Micronesia, Fed. Sts. OR Micronesia OR Moldova OR Mongolia OR Morocco OR Myanmar OR Nicaragua OR Nigeria OR Pakistan OR Philippines OR São Tomé and Principe OR Senegal OR Solomon Islands OR Sudan OR Swaziland OR Timor-Leste OR Tunisia OR Ukraine OR Uzbekistan OR Vanuatu OR Vietnam OR West Bank and Gaza OR Zambia OR Zimbabwe OR India OR Papua New Guinea |
|  | **Upper-middle income countries** |
| **#4** | Albania OR Algeria OR American Samoa OR Argentina OR Armenia OR Azerbaijan OR Belarus OR Belize OR Bosnia and Herzegovina OR Botswana OR Brazil OR Bulgaria OR China OR Colombia OR Costa Rica OR Cuba OR Dominica OR Dominican Republic OR Equatorial Guinea OR Ecuador OR Fiji OR Gabon OR Georgia OR Grenada OR Guatemala OR Guyana OR Iran, Islamic Rep. OR Iran OR Iraq OR Jamaica OR Jordan OR Kazakhstan OR Kosovo OR Lebanon OR Libya OR Macedonia, FYR Malaysia OR Maldives OR Marshall Islands OR Mauritius OR Mexico OR Montenegro OR Namibia OR Nauru OR Paraguay OR Peru OR Romania OR Russian Federation OR Russia OR Samoa OR Serbia OR Sri Lanka OR South Africa OR St. Lucia OR St. Vincent and the Grenadines OR Suriname OR Thailand OR Tonga OR Turkey OR Turkmenistan OR Tuvalu OR Venezuela, RB OR Venezuela |
| **#5** | #1 AND #2 |
| **#6** | #1 AND #3 |
| **#7** | #1 AND #4 |
|  | **Combined search** |
| **#8** | #5 OR #6 OR #7 |

**Supplementary File 2: Search terms used in Web of Science to identify studies on exposure to extremely low-frequency magnetic fields in LMIC**

| **Web of Science** | |
| --- | --- |
|  | **Exposure** |
| **#1** | Low frequency magnetic field OR ELF-MF OR ELF magnetic field |
|  | **Low income countries** |
| **#2** | (TS=(low frequency magnetic field OR ELF-MF OR ELF magnetic field) AND TS=(Afghanistan OR Benin OR Burkina Faso OR Burundi OR Central African Republic OR Chad OR Congo, Dem. Rep OR Congo OR Eritrea OR Ethiopia OR Gambia, The OR Gambia OR Guinea OR Guinea-Bissau OR Haiti)) |
| **#3** | (TS=(low frequency magnetic field OR ELF-MF OR ELF magnetic field) AND TS=(Korea, Dem. People's Rep. OR North Korea OR Liberia OR Madagascar OR Malawi OR Mozambique OR Nepal OR Niger OR Rwanda OR Sierra Leone OR Somalia OR South Sudan OR Syrian Arab Republic OR Syria Tajikistan OR Tanzania Togo OR Uganda OR Yemen, Rep. OR Yemen)) |
| **#4** | (#1 AND #2) OR #1 AND #3 |
|  | **Lower-middle income countries** |
| **#5** | (TS=(low frequency magnetic field OR ELF-MF OR ELF magnetic field) AND TS=(Angola OR Bangladesh OR Bhutan OR Bolivia Cabo Verde OR Cambodia OR Cameroon OR Comoros OR Congo, Rep. OR Congo OR Côte d'Ivoire OR Djibouti OR Egypt, Arab Rep. OR Egypt OR El Salvador OR Ghana OR Honduras OR Indonesia OR Kenya OR Kiribati OR Kyrgyz Republic OR Kyrgyzstan OR Lao PDR OR Laos OR Lesotho OR Mauritania OR Micronesia, Fed. Sts. OR Micronesia)) |
| **#6** | (TS=(low frequency magnetic field OR ELF-MF OR ELF magnetic field) AND TS=(Moldova OR Mongolia OR Morocco OR Myanmar OR Nicaragua OR Nigeria OR Pakistan OR Philippines OR São Tomé and Principe OR Senegal OR Solomon Islands OR Sudan OR Swaziland OR Timor-Leste OR Tunisia OR Ukraine OR Uzbekistan OR Vanuatu OR Vietnam OR West Bank and Gaza OR Zambia OR Zimbabwe OR India OR Papua New Guinea)) |
| **#7** | (#1 AND #5) OR (#1 AND #6) |
|  | **Upper-middle income countries** |
| **#8** | (TS=(low frequency magnetic field OR ELF-MF OR ELF magnetic field) AND TS=(Albania OR Algeria OR American Samoa OR Argentina OR Armenia OR Azerbaijan OR Belarus OR Belize OR Bosnia and Herzegovina OR Botswana OR Brazil OR Bulgaria OR China OR Colombia OR Costa Rica OR Cuba OR Dominica OR Dominican Republic OR Equatorial Guinea OR Ecuador OR Fiji OR Gabon OR Georgia OR Grenada OR Guatemala OR Guyana OR Iran, Islamic Rep. OR Iran OR Iraq OR Jamaica OR Jordan OR Kazakhstan OR Kosovo OR Lebanon)) |
| **#9** | (TS=(low frequency magnetic field OR ELF-MF OR ELF magnetic field) AND TS=(Libya OR Macedonia, FYR Malaysia OR Maldives OR Marshall Islands OR Mauritius OR Mexico OR Montenegro OR Namibia OR Nauru OR Paraguay OR Peru OR Romania OR Russian Federation OR Russia OR Samoa OR Serbia OR Sri Lanka OR South Africa OR St. Lucia OR St. Vincent and the Grenadines OR Suriname OR Thailand OR Tonga OR Turkey OR Turkmenistan OR Tuvalu OR Venezuela, RB OR Venezuela)) |
| **#10** | (#1 AND #8) OR (#1 AND #9) |
|  | **Combined search** |
| **#11** | #4 OR #7 OR #10 |
